# Copying and Evolution of Neuronal Topology

**DOI:** 10.1371/journal.pone.0003775

**Published:** 2008-11-20

**Authors:** Chrisantha Fernando, K. K. Karishma, Eörs Szathmáry

**Affiliations:** 1 MRC National Institute for Medical Research, Mill Hill, London, United Kingdom; 2 Collegium Budapest (Institute for Advanced Study), Budapest, Hungary; 3 Parmenides Foundation, Munich, Germany; 4 Institute of Biology, Eötvös University, Budapest, Hungary; University of Minnesota, United States of America

## Abstract

We propose a mechanism for copying of neuronal networks that is of considerable interest for neuroscience for it suggests a neuronal basis for causal inference, function copying, and natural selection within the human brain. To date, no model of neuronal topology copying exists. We present three increasingly sophisticated mechanisms to demonstrate how topographic map formation coupled with Spike-Time Dependent Plasticity (STDP) can copy neuronal topology motifs. Fidelity is improved by error correction and activity-reverberation limitation. The high-fidelity topology-copying operator is used to evolve neuronal topologies. Possible roles for neuronal natural selection are discussed.

## Introduction

This paper is the result of taking seriously the idea that units of selection exist in the brain [1]–[3]. A unit of selection is an entity that can replicate, and have hereditary variation [4], [5]. If these units have differential fitness they can evolve by natural selection. Examples of units of selection include: units of life [6] such as organisms and lymphocytes evolving by somatic selection [7], but also purely informational entities such as viruses, machine code programs [8] and binary strings in a genetic algorithm [9]. Since natural selection is an algorithm for generating adaptation [10], it can have many implementations [11]. It is worthwhile considering whether it may be utilized for cognition.

The theories of neural Darwinism [12] and neuronal selectionism [13], [14] propose that a primary repertoire of neuronal groups within the brain compete with each other for stimulus and reward resources. This results in selection of a secondary repertoire of behaviourally proficient groups [15]. Both Edelman and Changeux's groups have produced an impressive range of detailed models of hill-climbing type (exploration and exploitation) algorithms that can explain a wide range of behavioural and cognitive phenomena at various levels of abstraction [16]; such as category formation [12], reinforcement learning using spike-time dependent plasticity modulated by dopamine reward [17], visual-motor control in a robotic brain-based device [18], temporal sequence learning [19], effortful cognition in the Stroop task [20], and planning [21]. Importantly, both these research programs avoid the need for replication of neuronal groups, i.e. none of their algorithms require units of selection. This stimulated Francis Crick to distinguish Edelman's set of algorithms from the fundamental natural selection algorithm as defined, for example, by John Maynard Smith's formulation of units of evolution [22], [23]. At this stage, one might also mention Richard Dawkins' proposal of selective neuronal death as a memory mechanism, again a selectionist but non-Darwinian theory without need of replication [24].

It is crucial to be clear to what extent, if any, the algorithmic capacity of ‘natural selection’ to produce adaptation is limited if one removes the requirement for multiplication, and instead starts with a primary repertoire of solutions that compete for limited resources. We propose that the algorithms of Edelman and Changeux fundamentally consist of a population of stochastic hill-climbers [25]. Each neuronal group is randomly initialized, and those groups that are closest to a good solution obtain a greater quantity of synaptic resources allowing them to ‘grow’ and/or ‘change’. The critical assumption is that when those groups that are better at time *t* gain more synaptic resources, they are capable of transferring the functions that were embodied in their existing structures to the new substrate. Michod summarises the fact that in neuronal group selection, synaptic change rules replace replication as a mechanism of variability of the ‘unit of selection’: there is correlation between the parental and offspring states of the same neuronal group even without multiplication [26]. We contend that replication is the most natural (but not the only) way to envisage this transfer of function operation. Replication has the advantage of leaving the original solution intact, so that a non-functional variant does not result in loss of the original solution. Unless the neuronal group has the capacity to revert to its original state given a harmful variation, in which case it is effectively behaving as a 1+1 Evolutionary Strategy [27], there is the potential that good solutions are lost.

Furthermore, in evolutionary theory there is an emerging extended evolutionary synthesis [28] that addresses the issue of the evolution of evolvability, that is; how exploration distributions (the distribution of phenotypes that a given genotype produces) can be structured by evolution, to maximize the probability that a random genetic mutation produces a beneficial phenotype [29]. Although not without its critics [30], increasingly there is an understanding that natural selection is capable of acting self-referentially to improve itself as a heuristic search algorithm. For the evolution of evolvability to be possible, the unit of selection must encode the mechanism of its own self-replication. Not all units of selection are necessarily capable of this; however, a neuronal implementation may be ideally suited for this kind of self-referential encoding of a copying algorithm. It is for these reasons that we put forward a neuronal copying mechanism, capable of replication of neuronal group structure.

**Figure 1:**
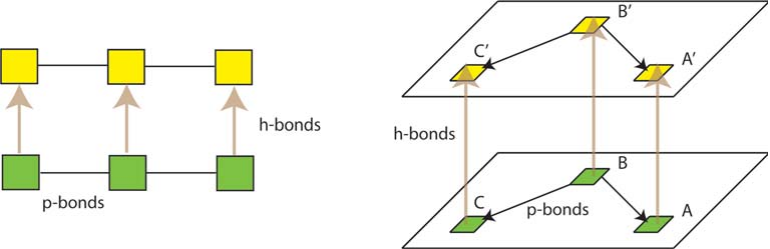
On the left is DNA replication, and on the right is neuronal replication. Green squares represent nucleotides and neurons of the parent. Yellow squares represent nucleotides and neurons of the offspring. Hydrogen bonds are equivalent to between-layer vertical connections, and phosphodiester bonds are equivalent to within-layer connections.

**Figure 2:**
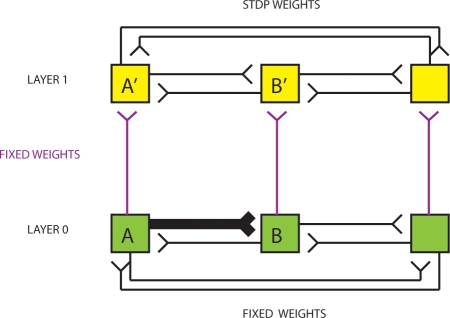
At one extreme, anatomical projections from L0 neurons to L1 neurons may be possible, such that a small-scale topographic map is obtained without the need for self-organizing algorithms. The single bold connection in L0 refers to a strong connection.

**Figure 3:**
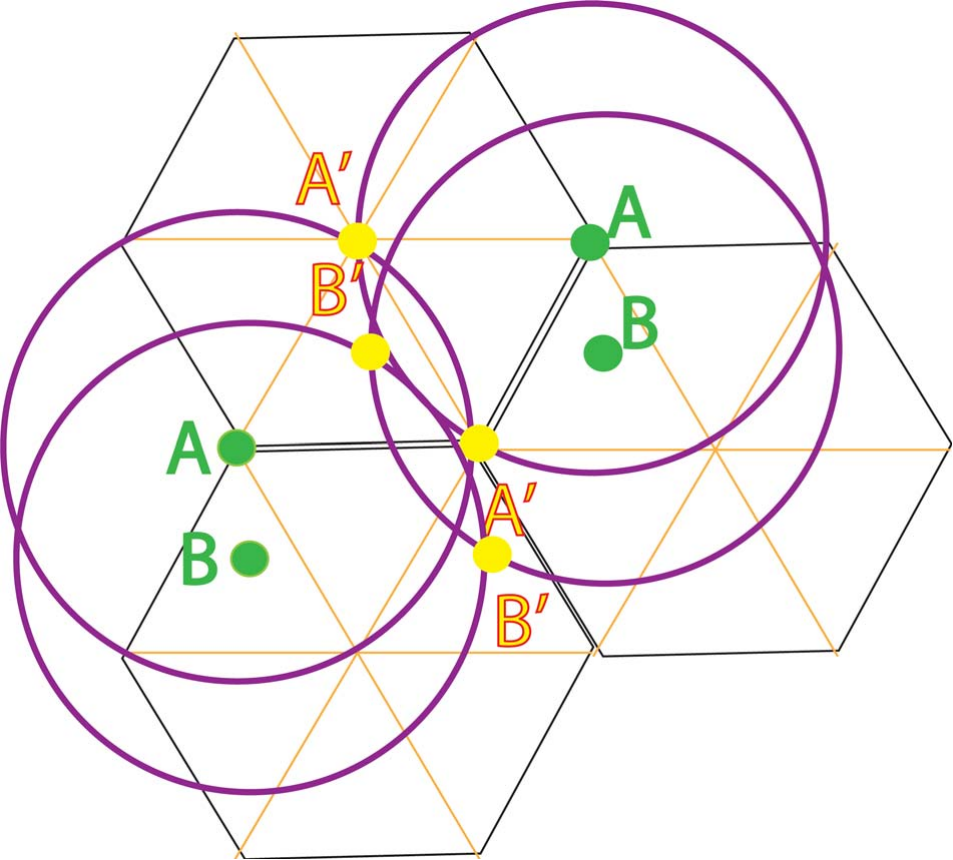
William Calvin proposes a hexagonal replicator on a single 2D surface layer of cortex. Each cell is a superficial pyramidal cell that has standard length collaterals that project in a circle of radius 0.5 mm. If two such neurons have the same receptive field properties and are separated by 1 mm, e.g. A and A, then these collaterals overlap at two points A′ and A′. The two neurons at A′ will receive superimposed correlated firing from the A neurons and will therefore ‘copy’ the firing pattern of the A neurons. Neurons displaced from A within the same triangle, e.g. B and B, will make similar copies, B′ and B′. However, this explanation does not explain how A's connections to B will be copied between A′ and B′, i.e. how ‘p-bonds’ are formed.

Explicit self-replication of neuronal groups has been proposed previously by William Calvin [31]; however, his mechanism only addresses half the problem; that of forming neuronal correspondences between parent and offspring networks (e.g. between A and A′ neurons in Figures 1, 2, 3), and not of reconstructing the parental synaptic topology within the child network. Figure 1 directly compares the genetic replicator system with our proposed neuronal replicator system. In the genetic system a parental DNA strand acts as a template for complementary activated nucleotides. Hydrogen bond (h-bond) formation between nucleotides is responsible for this association. Phosphodiester bond (p-bond) formation between the newly attached activated nucleotides re-creates the topology of connectivity found in the parental strand. William Calvin's model as we show later, achieves the equivalent of h-bond formation, but is not able to carry out the more difficult task of recreating the neuronal equivalent of the p-bonds, i.e. intra-layer connections. Note that in the neuronal case, reconstructing the pattern of p-bonds is a considerably more difficult problem than with DNA.

Another important contribution is that of Robert Aunger who takes a neuro-memetic viewpoint, claiming that neuronal organizations replicated within the brain prior to their ability to undertake inter-brain memetic transfer [32]. Although recent theoretical work hints at the possibility that linguistic constructions [33] may undergo natural selection dynamics in language acquisition [34], and may thus be one of the most plausible candidates for neuro-memes, no neuronal implementation has been proposed. Finally, the most explicit proposal so far of a neuronal unit of selection comes from Paul Adams who claims that synapses replicate and are neuronal units of selection, with mutations being noisy quantal Hebbian learning events where a synapse is made to contact an adjacent post-synaptic neuron rather than to enhance the connection to the current post-synaptic neuron [35]. Adams also proposes a mechanism for error correction, which we discuss later.

Here we present a computational model of the copying of higher-order neuronal units of selection (relative to Adam's synaptic replicators) at the neuronal group level. The unit consists of a neuronal assembly with a particular topology of excitatory connections. The mechanism of self-replication utilizes known neurophysiological mechanisms; namely, topographic map formation [36], [37], STDP [38] and neuronal resetting [39]. So far there has been no systematic search for evidence to suggest that these mechanisms in combination can achieve neuronal self-replication. Therefore, we explore the implications of what we call the *neuronal replicator hypothesis*, rather than providing confirmation of the hypothesis empirically. Thus our aim is to explore a self-consistent model that makes explicit what would be required for high-fidelity neuronal topology copying.

For a theory of evolution by natural selection, heredity is an indispensable element, and here we explore one mechanistic solution of neuronal replication that rests on connectivity copying of microcircuits. Note that this is not the only, and maybe not even the most plausible or even relevant solution. Even in organismic biology we have various systems for inheritance: inheritance based on metabolic networks, on conventional genes and epigenetic (including gene regulatory) mechanisms [40]. What matters for there to be units of evolution is that multiplication combined with heritable variation must somehow be solved. This can be done by connectivity copying of smallish circuits, but also with the transfer of activity patterns, an option explored elsewhere. The latter approach also allows for the possibility that indeed individual synapses or neurons may not matter too much, but groups of neurons with many synapses do.

The organization of the remainder of the paper is as follows. We present three models; each makes more assumptions about neuronal functionality than the last. We divide the problem of neuronal self-replication into two distinct parts: formation of a one-to-one topographic map between two neuronal layers, and reconstruction of the intra-layer topology of the parent in the offspring layer. To solve the first part we utilize the process of topographic map formation. We then examine the ability of two forms of spike-time dependent plasticity (STDP) to undertake intra-layer topology copying. Having observed that the exploration distribution of variants in both cases of STDP has high variance [29], i.e. that copying is of low fidelity, we present the second model that introduces two error-correction mechanisms. The first error correcting mechanism fixes false positive synapses, and the second type fixes false negative synapses. Although fidelity is increased, non-local activity spread results in the constant production of causal inference errors. The third model shows how non-local activity reverberation can be limited, thus allowing unlimited heredity of topology. Finally, we use the high-fidelity mechanism to demonstrate natural selection, discuss predictions of the neuronal replicator hypothesis and outline a research program to test the hypothesis empirically.

## Results

A range of potential solutions to the problem of neuronal copying exists, from anatomical (activity-independent) mechanisms, to self-organizing (activity-dependent) algorithms. The neuronal copying problem can be bisected as follows: the formation of neuronal correspondences between the parental and offspring networks (h-bonds in Figure 1); and the copying of the connection pattern present within the parental network, to the offspring network (p-bonds in Figure 1). By ‘parental’ and ‘offspring’ network we mean the original and the copied networks present in adjacent layers defined by a topographic map.

### Topographic Map Formation

**Figure 4:**
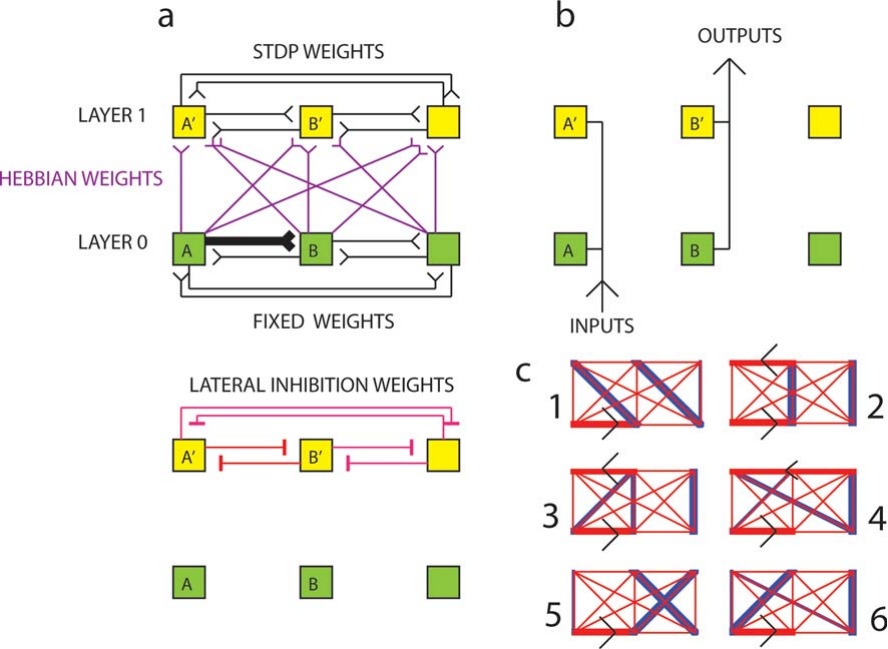
Part A (top) Formation of a topographic map using Hebbian learning and lateral inhibition. Assume that layer 0 (green neurons) project to layer 1 (yellow neurons) via initially all-to-all Hebbian Oja type neurons (purple). Weights in L0 are fixed (in this case only the weight from A to B is strong, all others are weak), and weights in L1 are plastic due to STDP. (bottom) There is all-to-all lateral inhibition in L1. Part B. A fixed topology I/O map with vertical correspondences is used to calculate the functions mapped by L0 and L1. Part C. “Shifts” and “Compression” are observed when the Hebbian learning+Oja rule+lateral inhibition algorithm is used in stochastic simulation using Izhikevich neurons [53]. See that the thick blue weights connecting L0 to L1 in Part C are not perfectly vertical, and neither are they always injective. In Part C(1) we see a “shift”, i.e. the representation of neuron B is shifted to the A′ position. Part C(5) and Part C(6) also contain shifts. However, in Part C(2) there is a perfect vertical mapping. In Part C(3) there is a “compression”. Compressions occur when the two (or more) parental neurons are highly correlated. In such a case, copying with compression may in fact be functionally beneficial in reducing information redundancy. In further experiments we assume perfect vertical strong connections.

Figures 2, 3, 4 demonstrate a range of known methods for forming a topographic map.

In Figure 2 we assume there is an anatomical mechanism for achieving perfect projections between parental and child neurons in each layer. That is, neuron A (B) in a parent layer will activate the spatially corresponding neuron A′ (B′) in the child layer.

Figure 3 shows another anatomical mechanism, proposed by Calvin for achieving one-to-one correspondence between neurons between the parent and offspring, this time based on a hexagonal organization of superficial pyramidal cells that possess a “doughnut” of activation [31]. Superficial pyramidal cells send a halo of excitatory connections laterally in a circle with radius 0.5 mm. Where the doughnuts overlap, the neuron at that site establishes the same receptive field as the parental neurons.

Figure 4 shows a self-organizing mechanism for topographic map formation. By utilizing Hebbian learning with Oja's synaptic renormalization rule [41] in the between-layer connections, and using lateral inhibition (soft-competition) in the child layer, it is possible to self-organize a topographic map between layers. The system starts with all-to-all connectivity from neurons in layer 0 to neurons in layer 1, see Figure 4 part A (top), and all-to-all lateral inhibition in L1, part A (bottom). Figure 4 part B shows a simple way in which inputs and outputs could be mapped between parent and child networks. We call this process I/O reallocation. Figure 4, part C shows the results of 6 randomly initialized simulations of topographic map formation using the above algorithm, see Methods for details. For subsequent models, for ease of analysis, we assume that some mechanism has been able to achieve a perfect one-to-one mapping between parental and child neuronal networks, as in Figure 2.

### Connectivity Copying

The second part of the copying operation is to re-construct within the child layer, the pattern of connectivity present in the parental layer, i.e. to make the p-bonds in Figure 1. Mechanism *A* (below) is the simplest one we investigated for achieving such copying. The weights in the parental layer (L0) are stabilized [17] . Strong perfect topographic mapping is assumed; vertical weights are assigned a fixed value of 20 mV+a random number drawn from a uniform distribution ranging between 0 and 10 mV.

Neurons in L0 are stimulated randomly at low frequency by external sources, e.g. at 5 Hz by Poisson spikes. Effectively, this noise acts as a set of low frequency, random external interventions [42]. If a strong enough weight exists between two neurons in L0 there will be fixed cross-correlation [43] between the firing of these neurons. In mechanism *A* only this cross-correlation data are used to allow L1 (the offspring layer) to infer the underlying pattern of connectivity in L0. Due to the topographic map between L0 and L1, neurons in L1 share a similar pattern of cross-correlation to neurons in L0; however, the weights between neurons in L1 are initially set to all be random and weak.

### Mechanism *A* for Connectivity Copying: STDP

The crucial baseline mechanism for copying is spike-time dependent plasticity (STDP) [37]. This is an asymmetric synaptic weight change rule that increments the weight between two neurons if a presynaptic spike arrives prior to the firing of the post-synaptic neuron, and decrements the weight if a post-synaptic spikes fires before a pre-synaptic neuron. In the absence of conduction delays and interference, this strategy should increment weights from causal neurons to effected neurons. We prevent weights from growing beyond an upper threshold that is set to 30 mV. In contrast to earlier work this means that just one pre-synaptic spike (rather than two) is sufficient to stimulate a post-synaptic cell [44], see Methods for details.

**Figure 5:**
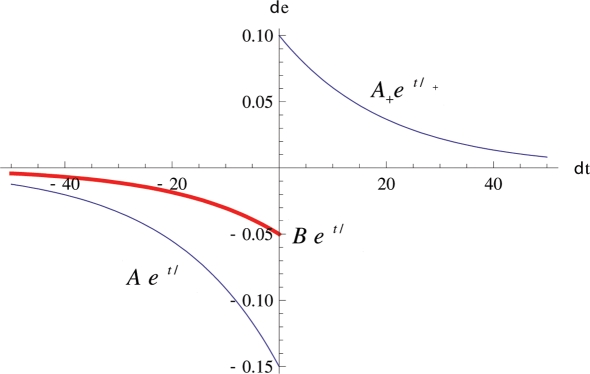
The STDP curves used. τ_+_ = τ− = 20 ms, A_+_ = 0.1, and A_−_ = 0.15, B_−_ = 0.05. The blue curves show the parameters used here and in [44]. The red curve shows the new LTD<LTP parameter B_−_ = 0.5 that we use here for more copying.

The phenomological STDP function is defined such that given uncorrelated pre- and post-synaptic spike trains, there will be a net decrease in synaptic strength. This is achieved by making the negative area of the function greater than the positive area of the function, see Figure 5. We use the parameters and model for STDP described by Eugene Izhikevich [44]. STDP is permitted only to modify the strength of weights between L1 neurons. L0 synapses are set to a particular configuration that we want to copy, and are held fixed, see Methods for details.

**Figure 6:**
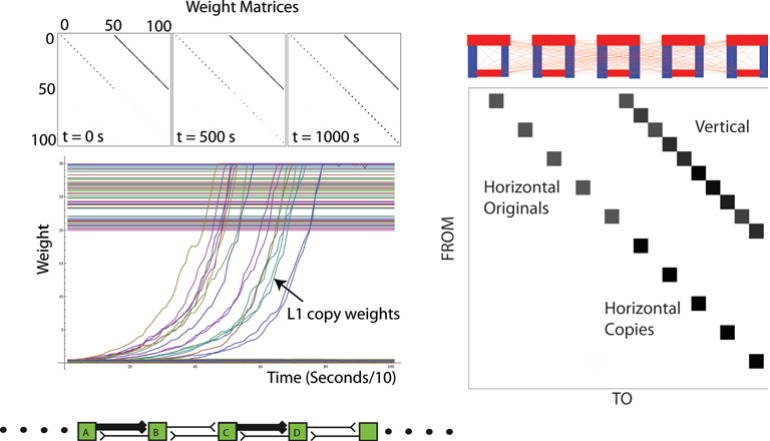
(Top Left) Weight matrices for two 50 neuron layers at the beginning, middle and end of the experiment. Weights in L0 are copied without error to L1. (Middle Left) Dynamics of weight change during the 1000 s experiment. Weights in L1 increase monotonically. (Bottom Left) A sample of the pattern present in L0. Strong weights pass from left to right, separated by weakly connected neurons. (Right) The weight matrix and corresponding view of the two layers at the end of an experiment with this time only 10 neurons in each layer. Again, copying is perfect, i.e. the red left-to-right connections (the neuronal equivalent of p-bonds) are copied perfectly from L0 to L1. Thick blue lines show fixed vertical connections from L0 to L1 (the neuronal equivalent of h-bonds).

Figure 6 shows that a binary sequence of excitatory connections of unlimited length could be copied with very high fidelity. The chain of neurons of length 50 (and 10, Figure 6, right) contained strong weights interspaced at regular intervals. The strong weights were always causally independent of each other, i.e. there were no neurons that were connected to two other neurons by strong weights. Structural copying was completely without error, as can be seen by examination of the weight matrix over time (Figure 6, top left). The duration of the experiment was 1000 seconds. However, whether sufficient weight change can occur within this period depends on the concentration of dopamine (DA), which in our experiments is set to a constant value of 0.3 µM, see Methods.

**Figure 7:**
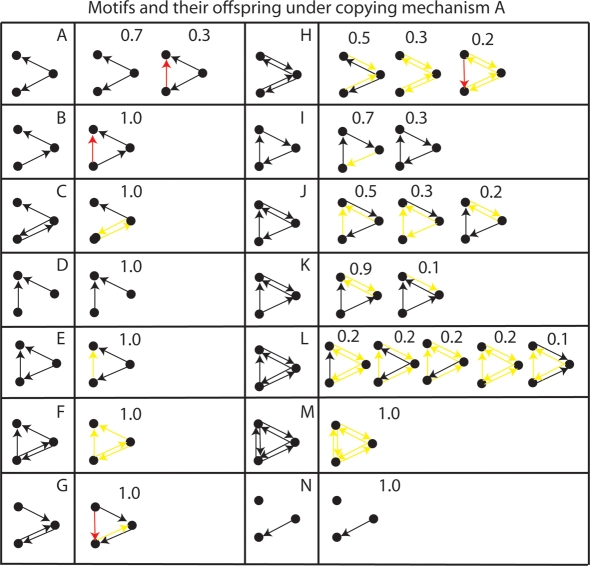
Motifs and the exploration distribution of offspring variants [29] resulting from copying by mechanism A. Red arrows show new connections, and yellow arrows show lost connections. The proportion of copies of each type is displayed above the copy structure. Neurons are referred to in the text as neuron 0 (top), neuron 1 (right), neuron 2 (bottom).

Figure 7 shows that mechanism *A* cannot copy all possible three neuron motifs without self-connections. We chose 3-node motifs because recent work has studied their prevalence in brain networks [45]. The systematic errors made by the copying mechanism are of four types:

#### 1. Mistaken Dependence

In motif A, neuron 1 causes 0 and 2 to fire, but 0 and 2 are independent conditional on 1. However, if there is an asymmetric delay in the influence of neuron 1 on neuron 0 and 2, then one will tend to consistently fire before the other. In this case, STDP will mistakenly infer that there was a direct causal link between 2 and 0.

#### 2. Transitive Inference

The new connection in motif B, exhibits the phenomena of transitive inference, i.e. because neuron 2 fires before neuron 0, a direct connection is made between 2 and 0, even though in the parental circuit, neuron 0 exerts its causal influence indirectly only via neuron 1.

#### 3. Reciprocal Interference

In motif C, neuron 2 connects reciprocally with neuron 1. Since STDP when neuron 1 fires and causes neuron 2 to fire will decrease the weight from 2 to 1 more than it increases the weight from 1 to 2, there will be a net decrease in weights in both directions. Mechanism A mistakenly interprets the neurons as independent of each other, when in-fact they mutually cause each other to fire.

#### 4. Causal Dominance

A direct causal process (if it is faster) may interfere with the copying of a slower indirect causal process. Motif E shows there are two pathways from neuron 1 to 0, direct, and indirect (via neuron 2). Neuron 0 is activated more rapidly by the direct pathway than by the indirect pathway. Since this activation results in neuron 0 firing before neuron 2, there will be a tendency for destructive interference with the real causal parental connection from 2 to 1.

The erroneous copying of other motifs can be interpreted using the above principles; for example in motif G, equivalence interference causes loss of a reciprocal connection, however, since neuron 1 is stimulated more often than neuron 2 (since it receives inputs from neuron 0), the reciprocal interference is asymmetric, with the weight from neuron 1 to 2 winning out. Once the pathway 0→ 1 → 2 has been established, then transitive inference establishes a direct connection from 0 → 2.

Motif H tends to form a common-cause motif since neuron 1 has the highest activation level because it receives 2 inputs whereas neurons 0 and 2 only receive 1 input each, i.e. asymmetric reciprocal interference is the explanation.

The loop in Motif I loses one of its links 70% of the time due to a special kind of causal dominance, i.e. neuron 2 causes neuron 1 to fire *via* neuron 0. Because this indirect route causes neuron 1 to fire after neuron 0, it results in interference with the real causal link from neuron 1 to neuron 2.

**Figure 8:**
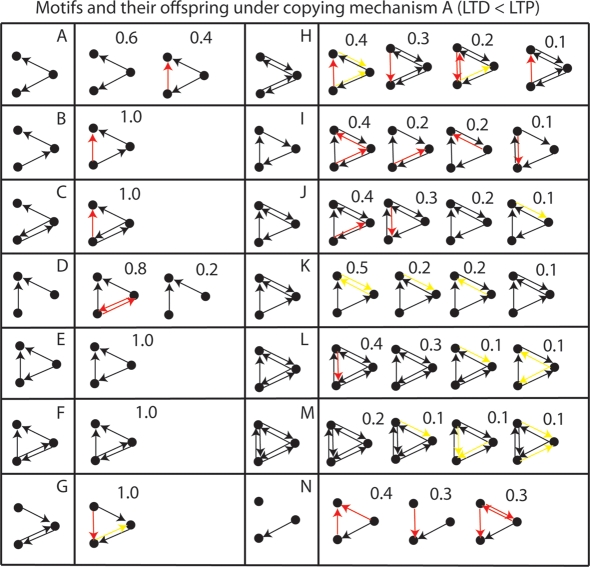
Motifs and the exploration distribution resulting from copying by mechanism A with modified LTD/LTP ratio. τ_−_ = 10 ms. Red arrows show new connections and yellow arrows show lost connections. The proportion of copies of each type is displayed above the copy structure.

The capabilities of mechanism *A* were tested with various modifications to the algorithm. Figure 8 shows the result of modifying the ratio of (long-term depression) LTD to LTP (long-term potentiation) by decreasing τ_−_ from 20 ms to 10 ms, resulting in a smaller negative area under the STDP curve. In contrast to figure 7, new red arrows (false positive connections) dominate over lost yellow arrows (false negative connections). Some motifs are copied perfectly which were not copied at all before, e.g. Motif F and E.

We remark here that the STDP function observed experimentally is closer to this second formulation, i.e. EPSP amplitude decrease with negative interspike intervals is not as large as the EPSP amplitude increase observed with positive interspike intervals [46]. Next, we examine two error correction mechanisms that compare the “phenotypes” of the two networks, i.e. the spike timings, and make directed changes to the “genotypes”, i.e. the underlying topologies. This is in contrast to error correction in DNA replication where only the genotype is checked for errors.

### Mechanism *B* for Connectivity Copying: Error Correction Mechanisms

**Figure 9:**
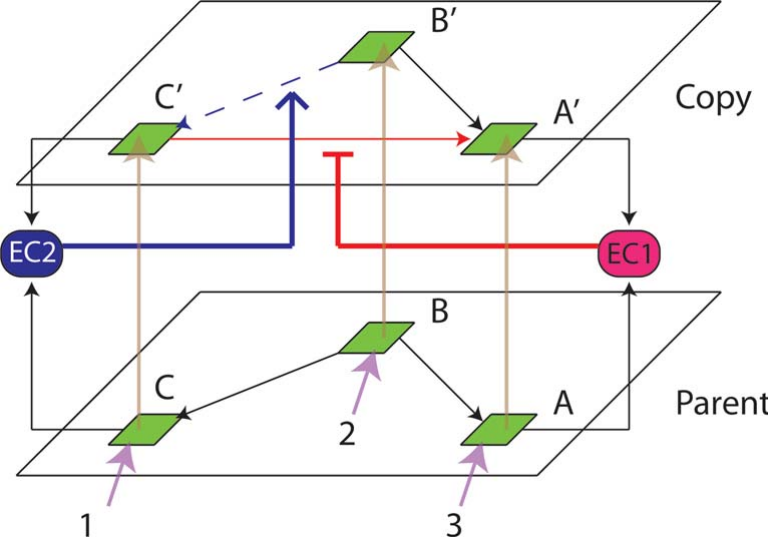
False positive error correction mechanism implemented using ‘observer’ neurons (EC1) that negatively neuromodulate neuron A′ in the copy layer on the basis of differences in firing between the parental (A) and copy (A′) layer neuron. We assume C is undergoing stimulation (1) when EC1 acts. False negative error correction mechanism (EC2) implemented using ‘observer’ neurons that positively neuromodulate inputs that pass to a poorly firing neuron (C′) in the copy layer from the neuron that is undergoing interventional stimulation (in this case we assume B is undergoing stimulation (2)) when EC2 acts. EC1 and EC2 type neurons are required for each neuron pair, A, A′, B, B′ and C, C′ and their neuromodulatory outputs must pass *widely* to all synapses in the child layer.

Mechanism *B* adds error correction to mechanism *A* by detecting neuronal spike-mispairing between layers and modifying afferent weights in the copy layer accordingly, see Figure 9. Error correction depends on modifying the child network based on observed differences between its activity and the parental network's activity. These methods attempt to remove false positive connections (red arrows, Figure 7–8) and increase weights where there are false negative connections (yellow arrows, Figure 7–8).

**Figure 10:**
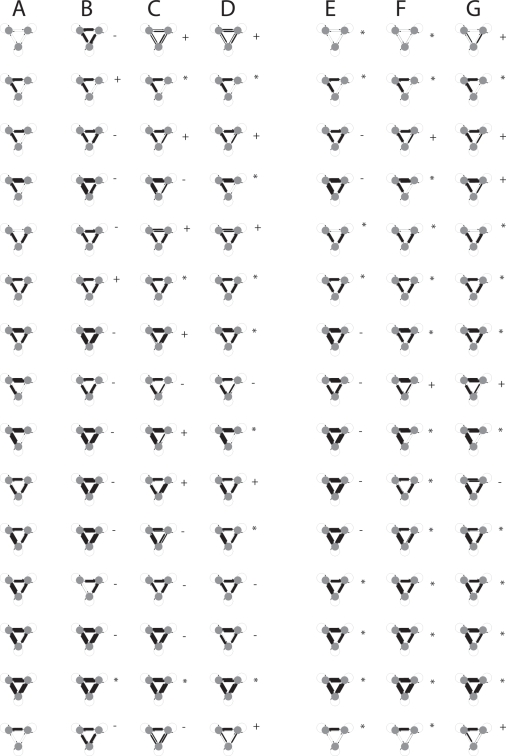
Typical offspring motifs produced by Mechanism A (column B) when copying parent motifs (column A). Read the figure by comparing the motifs in each column with the parent motif on the left column. Error correction mechanisms are introduced (EC1 in column C & EC1+EC2 in column D). Sparse activation is introduced in columns E, F and G. (Mechanism A = E, Mechanism B with EC1 only = F, Mechanism B with EC1+EC2 = G) Part A. Parental motifs. Part B. Control case. LTD<LTP type STDP with no error correction. Part C. As above but with error correction type 1. EC1 T = 10 ms. Part D. As above but with both types of error correction in use. EC1 T = 10 ms+EC2 S = 5 ms, ε = 0.01. Part E. Sparse activation. Control case. Part F. Sparse activation. EC1 T = 10 ms. Part G. Sparse activation. EC1 T = 10 ms+EC2 S = 5 ms, ε = 0.01. * = accurate copy, + = ‘semi-accurate copy’, − = erroneous copy (total error >30 mV).

Figure 10 shows the result of introducing the false positive error correction described in algorithm EC1 (figure 9) to both STDP copying algorithms previously studied. Figure 10a (left) shows the parental motif from which a copy is made (15 motifs are shown going down the column), 10b shows a typical offspring produced by Mechanism *A* without any error correction, and 10c shows a typical offspring produced by Mechanism *B* with EC1.

### Error Correcting Algorithm EC1 (See figure 9)

1. An observer neuron EC1 fires if A′ fires whilst A has not fired in the previous *T* (typically 10) milliseconds.
2. Neuron EC1 sends neuromodulatory efferents to A′ resulting in a decrease of synaptic eligibility traces of neurons afferent upon A′ in proportion to *φ* (typically 4) times their instantaneous eligibility traces.

This error correction system has the effect of punishing the neurons that caused A′ to fire when A did not fire. However, we see that when this error correction mechanism is used there is a tendency for the production of occasional false negatives (Figure 10c). This tendency can be tuned by adjusting a crucial parameter; the time period, *T*, within which A must have fired before A′ fires for there to be no down-regulation of afferents to A′. If *T* is too long then there is insufficient down-regulation, and if it is too short there is too much down-regulation, thus biasing the exploration distribution of variants [29]. By setting *T* = *10 ms*, EC1 could improve the fidelity of copying compared to using LTD<LTP STDP alone, see figure 10 part C (compared to part B which shows Mechanism *A* without EC1).

The second kind of error correction, EC2, involves enhancing inputs to a child neuron that is inactive when its parental counterpart is active. For example, Figure 9b shows that when neuron B is activated (by stimulus 2), that neuron C′ is under-activated relative to C. The EC2 neuron would then increase the weights of all synapses afferent upon C′, which includes the synapse from B′.

### Error Correcting Algorithm EC2 (See figure 9)

1. An observer neuron EC2 fires if C fires *and* C′ does not fire at least *S* (typically 5 ms) after C.
2. Neuron EC2 sends neuromodulatory efferents to C′ resulting in an increase of synaptic eligibility traces of all synapses afferent upon C′ by a small fixed increment *ε* = *0.001*.

Figure 10 Part D shows the significant improvement in copying obtained by the introduction of EC2 compared to using only EC1. See Methods for a justification for EC1 and EC2.

Next we examined the effect of sparse activation by reducing the background random thalamic input to 1 Hz. This is consistent with the spontaneous firing rate of neocortical pyramidal neurons [17]. Figure 10 columns E, F & G show the control, EC1 and EC1+EC2 copying mechanisms with sparse activation. Sparse activation considerably improves the fidelity of copying, even without reducing the time available for copying. The copies in Figure 10 Part G show the greatest similarity to their parental motifs. Sparse activation is beneficial for copying because it limits the extent of cross-correlations arising from non-causal associations, i.e. correlations due to the random associations in external stimuli that activate the parental layer, rather than causal relations produced by the parental layer itself.

**Figure 11:**
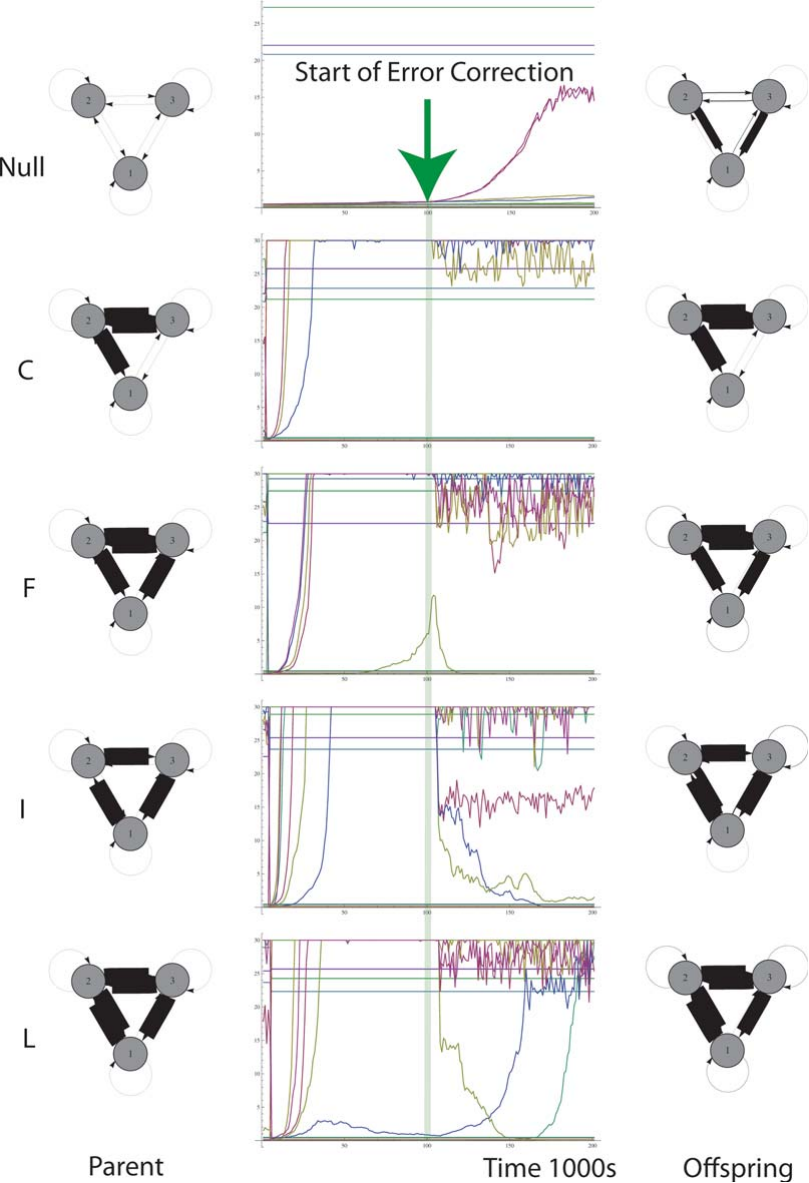
5 Motifs (Null, C,F, I & J) and their copying dynamics. Note that after error correction begins the weights change significantly. Note some zero weights in the parent stabilize at intermediate levels in the offspring. These intermediate level weights correspond to low probability of a pre-synaptic spike producing a post-synaptic spike. The tendency to produce these intermediate level weights can be tuned by adjusting the ratios of EC1 to EC2.

Figure 11 shows the dynamics of copying with Mechanism *B*, for a sample of motifs. Copying takes 1000 seconds in all cases, at which point the child motif is complete.

**Figure 12:**
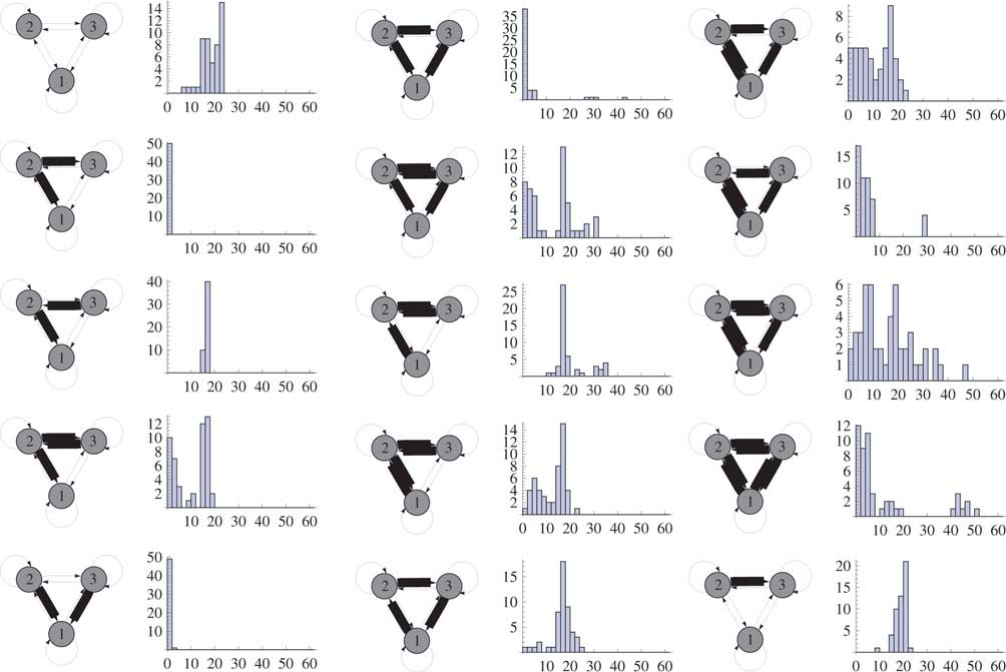
The 15 parental motifs are shown on the left of the histogram of the distribution of Euclidean distances of 40 offspring from itself. A Euclidean distance of 30 corresponds to a total weight difference equivalent to one maximal weight (30 mV).

Figure 12 shows the distribution of copying fidelity produced by Mechanism *B*. We define fidelity simply as the Euclidean distance between corresponding weights in L0 and L1. A Euclidean distance of 30 corresponds to a total difference of one maximal weight over all 6 weights.

A criticism at this stage is that copying fidelity is extremely poor. Mechanism A (STDP alone without error correction at 5 Hz activation, figure 10B) could only copy 2 out of 15 3-node motifs correctly, and Mechanism A with sparse (1 Hz) activation (figure 10E) could only copy 8 out of 15 motifs correctly. For mechanism B (Figure 10D) with 5 Hz activation only 7 of 15 motifs are accurately copied, 5 motifs were semi-accurately copied (“semi-accurate” here means connections that should have had a low or high weight instead exhibited a moderate weight), whilst 3 motifs were inaccurately copied. For mechanism B (Figure 10G) with sparse (1 Hz) activation, the accuracy of copying is improved compared to Figure 10D). The main type of error is the production of weak weights. Figure 11 shows this more clearly, e.g. motif NULL and I possess weak weights that are half the strength of the maximum weight. These weights are determined by the parameters of the EC1 error correction mechanism. A stronger error correction mechanism results in smaller steady-state weak weights. One pre-synaptic spike with a synaptic conductance of 20 mV is insufficient to produce a post-synaptic spike. Furthermore, adjustment of EC2 and EC1 mechanisms may be expected to improve fidelity. These error correction mechanisms are admittedly high in parameters and it is likely they are not the optimum settings for copying of all topologies. For example, with sparse activation EC2 actually makes copying less accurate, compare Figure 10G (with EC1) with Figure 10F (without EC1).

Figure 12 is evidence that copying rarely makes an error of more than one maximum weight (30 mV). If all weights were copied incorrectly the error would be 180 mV, and with random copying the error would be 60 mV on average. Some of the distributions of fidelities are multimodal, e.g. for the fully connected topology. Some motifs are copied perfectly, e.g. the fan-in motif (D) and the fan-out motif (A) whereas others are never perfectly copied, e.g. the chain motif (B).

### Mechanism *C* for Connectivity Copying: Activity Reverberation Limitation

To summarise, Mechanism A had poor fidelity for copying neuronal topology from one layer to another layer. It worked by first establishing a topographic map between the parental and the offspring layers. Spike-time-dependent plasticity in the offspring layer was then used to infer the underlying topology of the parental layer, on the basis of activity received from the parental layer neurons as they were randomly activated. To improve copying fidelity of neuronal topology, error-correction mechanisms were hypothesised that measured the difference in activity between corresponding neurons in parent and offspring layers. On the basis of this discrepancy of activity, they modified the afferents to the offspring neuron accordingly. Two types of error correction neuron were proposed to be required, false-positive and false-negative error correctors.

Both mechanism A and mechanism B show imperfect copying and extend poorly to arbitrary topology networks greater than 3-node motifs. This was because activity reverberation (re-entry) occurs to a greater extent in larger networks. When stimulating one neuron in layer 0, activity can spread over a wide range of L0 and L1, and this allows the four types of causal inference error previously described, i.e. mistaken dependence, transitive inference, reciprocal interference and causal dominance. These non-local cross-correlations mean that many possible underlying topologies can account for the phenomena. Activity spread is a particular problem due to the high synaptic conductances used.

**Figure 13:**
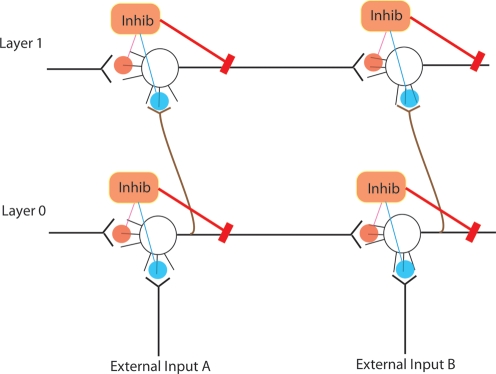
A reverberation limitation mechanism. Layer 0 is the parent layer that receives external random input at 1–4 Hz. Layer 1 is the offspring layer whose intra-layer synapses undergo STDP. Consider the case where external input A depolarizes the soma sufficiently to produce a spike. Since I_e_>I_I_, i.e. since the inputs from outside the layer are greater than the inputs from inside the layer, output spikes are sent along both axon collaterals. This results in activation of the neuron above, and the neuron to the right. The neuron to the right will experience depolorization, and STDP at the intra-layer synapses, however, the spike will be inhibited from passing to further intra-layer neurons because I_i_>I_e_ in this case. The inhibitory neuron associated with each excitatory neuron is responsible for implementing the rule ‘if *I_i_/I_e_*>*θ* then block intra-layer spike transmission’.

Mechanism C is a modification added to Mechanism B that prevents this kind of activity reverberation, see Figure 13. During the copy procedure, recall that only layer 0 is stimulated with one spike at a time at frequency 1–4 Hz. Assume that the source of depolarizing current to each neuron can be classified as either intra-layer, *I_i_*, (from afferent neurons within the same layer), or inter-layer, *I_e_*, (from afferents outside the layer). If *I_i_/I_e_*>*θ*, where θ = 0.1, then the post-synaptic neuron does not send a spike to neurons within the same layer, but does send a spike to neurons in other layers (i.e. passes the signal vertically but not horizontally). This ensures that if most of the current causing the neuron to fire is from a neuron within the same layer, the post-synaptic neuron does not pass this signal onto other neurons within the same layer, but only along vertical fibres to neurons in other layers. Despite this we allow STDP to occur at all intra-layer synapses onto the post-synaptic neuron. On the other hand, if the current is mainly external, then a spike is produced that passes to both intra-layer and inter-layer neurons. The effect of this modification is to force only *local horizontal spread* of activation, but allow *global vertical spread*. This eliminates causal inference errors that are made due to non-local correlations, and allows larger networks to be copied.

In Figure 13 we show that an associated inhibitory neuron undertakes this classification operation by sending dendrites to contact the intra- (red) and inter- (blue) layer afferents to the excitatory post-synaptic neuron. If the inhibitory neuron is activated then it blocks the intra-layer spike from passing to the downstream intra-layer neuron, but allows the spike to pass to the corresponding neuron in the child layer. We assume the inhibitory neuron can calculate the function ‘if *I_i_/I_e_*>*θ* then fire’, see Methods for details.

To summarise, the crucial requirement to copy large networks is a phase without external sensory stimulus in which (i) depolarisation of the neuron can be categorised as coming from either intra-layer or inter-layer afferents and, (ii) collateral (output) gating can limit the outgoing spike to inter-layer collaterals on the basis of this categorisation. This produces local spread within a layer, but allows unhindered spread of activity along topographic collaterals.

**Figure 14:**
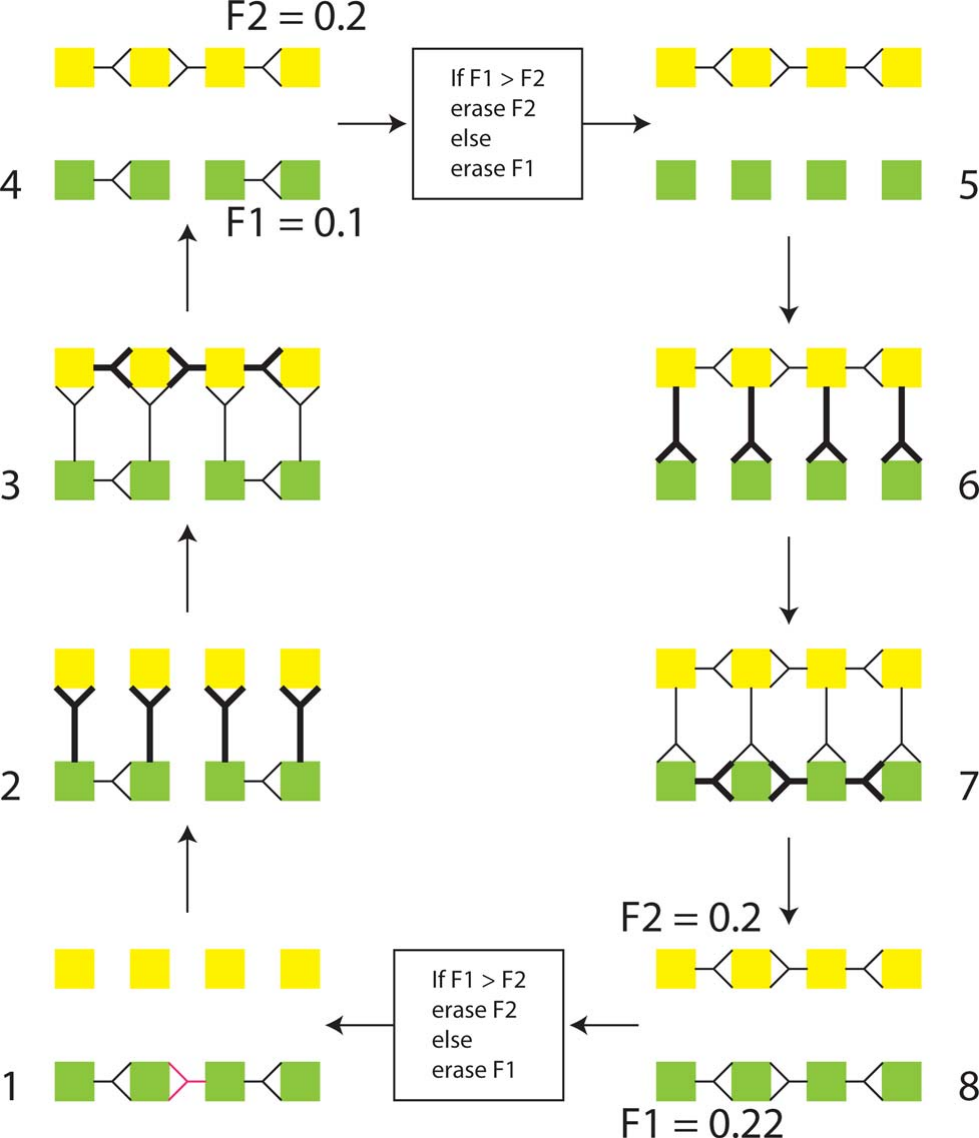
A neuronally implemented 1+1 ES using the STDP based copying mechanism. 1. The circuit to be copied exists in the lower layer L0. The black connections in L0 show the original circuit. 2. Horizontal UP connections are activated, e.g. by opening neuromodulatory gating. These are the equivalent of the h-bonds in DNA copying. 3. A copy of the topology of L0 is made in L1, using STDP and error correction. 4. The layers are functionally separated by closing neuromodulatory gating of the UP connections. The fitness of each layer is assessed independently. 5. The layer with the lowest fitness is erased or reset, i.e. strong synaptic connections are reduced. In the above diagram we see that L1 fitness >L0 fitness, so L0 experiences weight unlearning. 6. DOWN vertical connection gates are opened. 7. STDP in layer 0 copies the connections in L1. 8. After DOWN connections are closed, fitness is assessed and the cycle continues.

Mechanism C is sufficiently accurate that we can use it to evolve desired topologies in a neural implementation of a 1+1 evolutionary strategy (ES) [27] as shown in Figure 14. A 1+1 ES is a simple evolutionary algorithm that works as follows. If the offspring (yellow in parts 1,2,3 and 4) does not have fitness higher than the parent (green in parts, 1,2,3 and 4), then the offspring is erased and another attempt at copying the parent can be made (not shown). If the offspring has fitness higher than the parent, then the parent is erased and the offspring becomes the new parent and makes a new offspring in what was previously the parental layer, (see parts 5,6,7,8).

Note that neuromodulation is critical in the function of the 1+1 ES circuit in three ways. Firstly, the direction of copy making depends on modulation to open and close vertical up and down gates at different times. Gating of all types is a subject of recent intense interest [47]. Secondly, neuromodulation is necessary to switch on and off STDP based plasticity in L0 and L1 alternatively, this has been discussed above. Thirdly, a mechanism is necessary to *reset* the layer (i.e. reduce weights in the layer) that is to be overwritten. As discussed by Francis Crick, this may be one of the functions of deep sleep [39], [48].

Because copying using mechanism C has high fidelity, explicit mutation operators had to be introduced. After copying, each offspring layer receives one new random synapse, with an initial random weight from the range 0 to 30 mV. This “mutation operation” does not depend on errors intrinsic to the copy operation, but is externally imposed. In reality, mutation would also occur due to errors in the topographic map.

### Evolution of 6-Node Neuronal Topology

**Figure 15:**
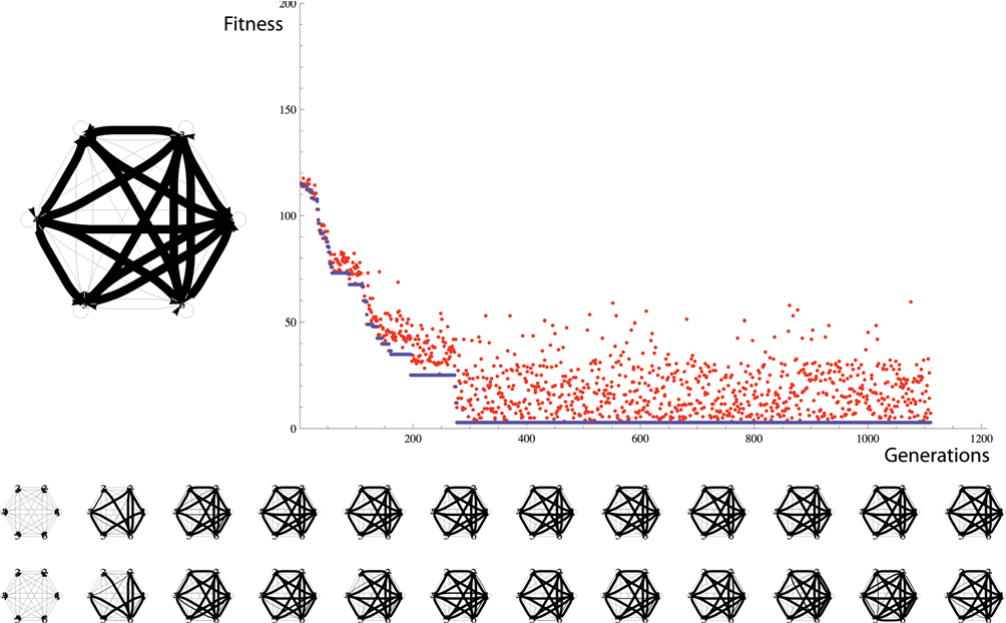
(Top Left) desired topology. (Top Right) Fitness vs. Generations. (Bottom) 12 examples of parent (top) – offspring (bottom) pairs. The neuronal copying algorithm is capable of sustaining evolution by natural selection to optimize the topology of a 6-neuron topology motif. The desired topology is shown on the top left. The 12 pairs of graphs show the parent (top) and offspring (bottom) networks at 100-generation intervals. The initial topology was fully connected by weak weights. The blue line in the fitness graph shows the fitness of the parent, and the red dots show the fitness of the offspring. The relationship of the blue line to the red dots shows that fitness (as defined here) is heritable for most configurations.

Here we initialise the parental and offspring 6-node network layers with weak all-to-all connections, i.e. within each layer, neurons are fully connected with random weights in the range 0 and 1 mV in magnitude. This corresponds to a “genome size” of 30 synapses. Between layers, strong collaterals connect the parent to the offspring network in perfect one-to-one vertical correspondence. Collateral weights are randomly initialized between 20 and 30 mV, i.e. they are all strong. A desired topology in the parental layer (L0) is generated by randomly choosing 50% of weights to be strong. Figure 15 shows an example evolutionary run in which the fitness function is the Euclidean distance to this desired 6-node topology. Note that within 300 generations a topology very close to the desired topology has evolved.

### Evolution of 10-Node Neuronal Topology

**Figure 16:**
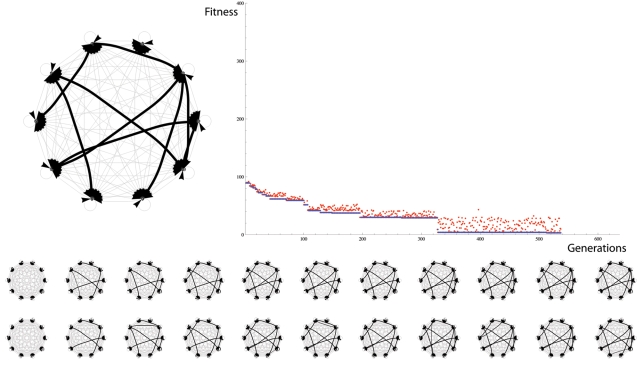
(Top Left) desired topology. (Top Right) Fitness vs. Generations. (Bottom) 11 examples of parent (top) – offspring (bottom) pairs. The neuronal copying algorithm is capable of sustaining evolution by natural selection to optimize the topology of a 10-node motif. Desired topology on the top left (randomly initialized with 10% connectivity). Fitness (Euclidean distance between desired and actual topology) of parent (blue) and offspring (red) over 600 generations. Bottom graphs shows 11 parent-offspring pairs taken at intervals of 50 generations.

Figure 16 shows an example evolutionary run in which a 10-node neural network is evolved using the same copying algorithm. This corresponds to a “genome size” of 90 synapses.

In both cases the desired topology was evolved successfully.

## Methods

The topology of our network consists of two layers. Each layer is a fully connected three-neuron network (without self-connections). Layer 0 projects to layer 1 by vertical topographic connections, see Figure 1. In all experiments apart from those shown in Figure 3 we assume the vertical weights are strong and fixed, set to 20 mv+rand(0,1)×10 mV. rand(0,1) is a floating point random number drawn from a uniform distribution with range between 0 and 1.

Are we justified in assuming very strong synaptic strength between and within layers so that on average one spike in the pre-synaptic neuron is sufficient to produce one spike in the post-synaptic neuron? Certainly such an assumption would seem unwarranted in light of Abeles' argument that “In the cortex, reliable transmission of activity is possible only between populations of cells connected by multiple diverging and converging connections.” (p210) [49], which is based on experimental calculations of asynchronous synaptic gain (ASG) that is the probability that a spike occurs in the post-synaptic neuron after a spike in the pre-synaptic neuron, over and above background spiking levels. Cortico-cortical connections typically have ASGs of 0.003 going up to 0.2 at best (p102 *ibid*), although thalamo-cortical connections have higher ASGs. Using the synaptic gains assumed by Izhikevich, at least 2 pre-synaptic neurons must fire close together for a post synaptic neuron to fire. However, recent work by Alain Destexhe's group has shown that high-conductance states, i.e. where neocortical neurons are subject to intense synaptic bombardment, can result in enhanced responsiveness and gain modulation, i.e. there can be a response even to single presynaptic spikes [50]. Furthermore, it appears that the “up” state of slow wave sleep is a high-conductance state [51], a finding that is parsimonious with the finding that sleep improves the human ability to solve insight problems. We have proposed elsewhere that insight problem solving may require heuristic search by natural selection [52]. In addition, within Izhikevich's framework several mechanisms exist for increasing the sensitivity of a neuron to a single spike, for example, using a mixed mode neuron described by the following parameters (*a* = *0.02*, *b* = *0.2*, *c* = *−55*, *d* = *4*, *I* = *2*), a single EPSP of 10 mV is sufficient to cause a post-synaptic spike [53].

Weights in both layers are initialized to rand(0,1)×0.5. Only in the experiments shown in Figure 3 we assumed the vertical weights were modified by Oja's rule [41] with Hebbian learning rate (η) 0.5, and weight decay rate (α) 0.002, plus lateral inhibition mediated by winner take all inhibitory spikes of −30 mV emitted to all excitatory neurons in Layer 1 by interneurons connected to any neuron that fired in Layer 1. The intention was that Oja's rule would prevent many-to-one connections from Layer 0 to Layer 1, and that lateral inhibition would prevent one-to-many connections from Layer 0 to Layer 1, thus producing a 1-to-1 topographic map.

Note that in Figure 3 there are still shifts and compressions at the neuron-to-neuron level. This can be abolished by assuming hard competition (winner-take-all dynamics) as is standard in many models [25], [54]. STDP models have indeed been shown to be capable of refining topographic maps [37] (see Figure 6 of that paper), and recently it is observed that the gamma cycle can implement a rapidly repeating winner-take-all algorithm [55]. Adams has also proposed the existence of topographic maps refinement systems in corticothalamic loops [35], [56]. Despite these precedents it is still correct to question the difficulty of obtaining a perfect topographic map, to which we can provide two responses.

Firstly we believe microscopic topology, although difficult to observe, is fundamental to neural function. Unfortunately, we do not yet know the details of local microcircuitry at the resolution of single neurons for most regions of the brain. A widely held view is that “human cognitive functions depend on the activity and coactivity of large populations of neurons in distributed networks” [57], however this does not logically imply that single synapses cannot influence behaviour. Although “alterations of single synapses or cells have not been shown to have similar macroscopic effects” to alterations in single base-pairs [57] this metaphor is misleading for the reason that neither have alterations in single base-pairs been shown to affect ecosystem dynamics. Natural selection, like human creativity, is hard to predict. In arguing that we should not worry about microscale circuitry, Sporns et al (2005) say that “individual neurons and connections are subject to rapid plastic changes” such as changing synaptic weights, structural remodelling of dendritic spines and presynaptic boutons, and “switching synaptic connections between large numbers of potential synaptic sites” [57]. However, we suggest it is these very features, i.e. “the vast number, high variability, and fast dynamics of individual neurons and synapses” that render them appropriate as basic neural information processing elements in a natural selection algorithm. Indeed where we have been able to explore microcircuitry, we have found that an almost perfect one-to-one *microtopographic maps* exists. David Marr writes that in the cerebellum “Each climbing fibre makes extensive synaptic contact with the dendritic tree of a single Purkinje cell (p), and its effect there is powerfully excitatory.” [58]. Other examples of high resolution topographicity include cortical layer 6 neurons that project with small terminals to the some thalamic nuclei [59]. Therefore, although we accept that perfect topographicity is not ubiquitous, there is evidence for it in some brain regions and therefore it is a reasonable basis for a model at this exploratory stage.

Our second response is that a perfect topographic map is not necessary for neuronal topology copying. We emphasize that the assumption is only made for the ease of analysis by giving the following example. Assume that neuron *A* in layer 0 has a receptive field that represents stimulus *A* and Neuron *B* in layer 0 produces response *B*. Imagine that neuron *A* in layer 0 projects to neuron *B* in layer 0. That is, the *function* of layer 0's neural topology constitutes the stimulus-response relation “stimulus *A* causes response *B*”. Now imagine an *imperfect topographic map*. Here, instead of neuron *A* mapping to neuron *A′* in layer 1, it maps to neuron *C′* in layer 1. Similarly assume that neuron *B* in layer 0 maps to neuron *D′* in layer 1. STDP in layer 1 will now reconstruct the relation between *A* and *B*, but now displaced and embodied between neurons *C′* and *D′*, not between neurons *A′* and *B′*. Because in this paper we are interested purely in topology copying, we would have had to apply a graph isomorphism algorithm to the topologies in both layers in order to establish their similarity. There are an infinite number of ways of measuring the similarity of two graphs. No way is more justified than another without reference to function. By assuming perfect topographicity we can simply use Euclidean distance as a measure of the similarity between two node-labeled graphs. In reality, a selection algorithm would act on the function of a layer, *not* its topology. For the copy layer to have the same *function* as the parent layer, it follows logically that an input/output reallocation algorithm would have to be invariant to this translation. Alternatively, if response space and stimulus space are themselves topographically organized, an assumption for which there is an enormous amount of evidence, e.g. retinotopic maps, topotopic maps, somatotopic maps and motor maps [60] then an inexact mapping will represent a mutated stimulus-response function. Therefore, imperfect topographic mapping is one method for producing mutations. It is the combination of the extent of topographicity between layers, and the compositionality of the mapping of *function* to each layer by an I/O reallocation method that determines the fidelity of *function copying*. A further discussion of function copying is not necessary for the internal consistency of claims made here.

We use a neuronal and weight change model very similar to that used in [17]. We model two layers of cortical spiking neurons with spike-time-dependent plasticity (STDP) modulated by dopamine (DA) reward occurring only in the offspring layer L1. Weights in the parental layer L0 are fixed. In all experiments we hold [DA] fixed. The STDP function (Figure 4) determines weight change *indirectly*, by producing a synaptic tag or eligibility trace molecule. It is the interaction between this eligibility trace at each synapse and the global reward signal (DA) that results in weight change.

Are we justified in assuming that different synapses are subject to different weight change rules? There is considerable evidence that synapses vary in plasticity, and that the extent of plasticity is under the control of neuromodulatory neurons [61]–[65]. It is a standard assumption in many models that neuromodulation can alter learning rates [66], [67]. Furthermore, recent research suggests that pre-synaptic inhibition is synapse-specific [68], [69], and thus can define a “weight modulation” matrix which can allow “switching” between different subtasks that require different plasticity profiles in the circuit [70], e.g. copying alternatively from L0 to L1 and from L1 to L0. Abbott's group propose that synapses have distinct states of stability and plasticity [71]. This kind of process is called metaplasticity, “the plasticity of synaptic plasticity” [72], [73]. Therefore, we feel justified to assume that a neural mechanism could be capable of holding weights fixed in some layers whilst allowing weights in other layers to vary. Note also that fixed inter-layer weights are not critical. Although we do not model this process further in this paper, from research in evolutionary computation and computational neuroscience (e.g. the Leabra algorithm [74]) it is known that Hebbian learning can bias the copying operator or the hill-climbing operator so as to structure the exploration distribution of variants, thus potentially optimizing evolutionary search [29]. Finally, the extent of STDP can differ depending on the dendritic location of the synapse [75], suggesting that vertical connections may be prevented from undergoing STDP whilst horizontal connections may be subject to STDP, even in the absence of neuromodulation.

### Neuronal Model

All neurons were excitatory neurons of the regular spiking type [44], and were fully connected within layers. The spiking model is from Izhikevich's seminal paper [53]:
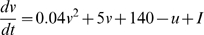
(1)

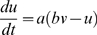
(2)with resetting after a spike as follows..
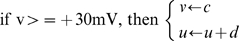
(3)
*v* represents membrane potential, and *u* represents a membrane recovery variable. When *v* reaches +30 mV (the apex of the spike, not to be confused with the firing threshold), *v* and *u* are reset. *b* = *0.2*, *c* = *−65*, *a* = *0.02*, *d* = *8*, corresponding to cortical pyramidal neurons with regular spiking. I is the input from other neurons, and external sources. Input was provided to one random neuron in layer 0 at a time, as an external activation (spike) of 17 mV being given with a probability ms^−1^ per layer of 0.02 (or 0.005 in the sparse activation regime). This resulted in a 5 to 1 Hz firing of neurons in layer 0. No direct external input was given to layer 1 neurons. The axonal conduction delay between intra-layer neurons was set to 1 ms for mechanism A, however, when EC1 and EC2 were introduced (Mechanism B), it was necessary to increase this intra-layer delay to 10 ms. Figures 6 & 7 were produced with 1 ms delays, and figure 9 was produced with 10 ms delays. Between layer delays were always set to 1 ms.

There is evidence for the kind of operations required by EC1 and EC2 mechanisms. For example, feedforward inhibition mediated by ‘fast-spike’ interneurons in layer 4 of neocortex could potentially implement the EC1 mechanism for abolishing false positives [76]. This system inhibits neurons in the cortex that do not fire synchronously with the corresponding neuron in the thalamus, and could thus have the same function as the EC1 neurons described above by preventing the firing of all but the corresponding neuron. Its effect would be indirect compared to the EC1 neuron because STDP would be responsible for the decay of weight from the inhibited neurons to the correct neuron. Furthermore, the complex pattern of inhibitory interneurons in cortex is poorly understood and could certainly support similar intra-cortical operations. Note that in evolution, the number of inhibitory neuron types has increased. Various activity-dependent heterosynaptic inhibitory modulation mechanisms are known [77], [78]. Although it is not known whether the topology of these circuits correspond to those presented here, their existence means that EC1 type mechanisms are not out of the question. There is less evidence for an EC2 mechanism; however, it is not *ad hoc* for it can utilize the same coincidence detection device as EC1 and needs only to potentiate rather than inhibit afferents to the post-synaptic neuron. Furthermore, independent of a natural selection algorithm, there are good reasons to believe that neuronal topology error-correction may be required in the brain. Abeles shows that due to neural death, 0.5 percent of cortical cells die at random every year. Almost all chains in a set of parallel independent chains of 100 cells will become inoperative. At least 60,000 parallel chains would be required to ensure at least one remains intact after 20 years (p210) [49]. The solution he proposes is the synfire chain consisting of converging/diverging connections between nodes where each node is between 5 and 360 neurons in size. Another solution one should at least consider is error-correction in which a broken chain is repaired by ‘observing’ the behaviour of other chains.

Reverberation limitation is implemented by assuming an associated inhibitory neuron can classify inputs as being intra-layer or inter-layer and gate an output collateral accordingly. We certainly do not exclude that there may be more ways of implementing this reverberation limitation operation. One interesting possibility is that synchrony by slow wave oscillations can gate activity in a directional manner [79]. This is particularly relevant given the recent finding that in prefrontal cortex, during slow oscillation, there is a fine balance between excitation and inhibition that “may allow for rapid transitions between relatively stable network states, permitting the modulation of neuronal responsiveness in a behaviourally relevant manner” [80]. Also, complex functions can be calculated within the dendritic tree itself [81]. More straightforwardly, a recent study has found that sparse activity in the excitatory neurons of layer 2/3 or layer 5 of the somatosensory cortex results in the recruitment of a recurrent inhibitory circuit consisting of inhibitory interneurons that are somatostatin positive. Through this mechanism, one pyramidal cell can inhibit an estimated 40% of its neighbours. When two pyramidal cells are spiking the resultant recurrent inhibition increases nonlinearly as a result of a tenfold increase in the recruitment of the inhibitory interneurons which receive convergent inputs [82]. Such a circuit would limit activity spreading in the horizontal layers, thus preventing reverberation, while the principal neuron continues to integrate information and convey it to the next processing region. The somatostatin neurons in question are activated only when one or a few pyramidal neurons are spiking. A circuit with similar properties occurs in the hippocampus [83].

### STDP Model

As described in [15] each synapse has two variables, a synaptic weight *w* and an eligibility trace *e*.
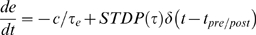
(4)

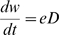
(5)where *D* is the extra-cellular concentration of DA in µM (which we always keep at 0.3 µM in all experiments) and δ(t) is the Dirac delta function that increases *e* by an amount specified by the STDP curve in Figure 2, although parameters differ depending on whether we choose to use the LTD>LTP variant or the LTP>LTD variant of STDP. τ = t_post_−t_pre_−delay_pre-to-post_, i.e. the interspike interval. If a pre-synaptic spike reaches the post-synaptic neuron (taking into account condition delays) before the post-synaptic neuron fires, then STDP(τ) is positive. If a pre-synaptic spike reaches a post-synaptic neuron after it fires, then STDP(τ) is negative. The eligibility trace *e* decays with time constant τ_e_ = 1 s. The synaptic weight *w* changes as the product of *e* and *D*. Weights were limited to a range from 0 to 30 mV.

The implementation of STDP is as follows. Every time a neuron fires, an STDP variable for that neuron is reset to 0.1. As described in [44] A.3, every millisecond timestep, STDP decreases by 0.95× STDP so that it decays exponentially according to 0.1e^−t/20 ms^, as in Figure 4. When a neuron fires, we increase the eligibility of each afferent synapse onto this neuron by an amount STDP(τ). Also, we make a data structure that represents the spike that was fired, and we track when it reaches each neuron that is efferent from the spiked neuron (taking delays into account again). When that spike reaches each of the efferent neurons, we decrement the eligibility by 1.5× STDP(τ). Note the co-efficient 1.5. This is the setting used in the LTD>LTP version of STDP. In the LTD<LTP version of STDP the co-efficient is 0.5. For computational efficiency, weight change is only undertaken every 1 s, by equation 5.

## Discussion

We proposed three increasingly accurate neuronal mechanisms for the copying of network topology from one region of brain to another. It is important to clarify that this is quite different from the STDP mechanism proposed to account for cortical reorganization of receptive fields due to deafferentation of sensory cortex [84]. Our mechanism allows *local circuitry and not just receptive fields to be copied between regions* (Figure 9). In contrast, William Calvin's neural ‘copying’ mechanism only explains how receptive fields are copied (Figure 2).

This contribution can be understood at several layers. First, it can be regarded as a purely theoretical exercise to demonstrate that, regardless of what actually is happening in the brain, one could use and tune known component processes of the brain to construct neuronal replicators. This interpretation allows for the possibility that, alas, the proposed connectivity-copying replicators in the brain do not exist. Second, our proposal can be regarded as a hypothesis on how parts of the brain actually work: we offer in this sections some arguments and facts that this interpretation is also legitimate. Third, the thoughts here can be regarded as stimulants for other ideas (including those resting on the transfer of activity patterns without connectivity copying) that might be more plausible or real.

We proposed a novel function for topographic maps; to act as the neuronal equivalent of h-bonds for copying operations. This adds to recent ideas formulated by Gary Marcus about the function of topographic maps in cognition [85]. We make the empirical prediction that our neuronal replicators should be found perpendicular to topographic maps outside sensory areas, for example perpendicular to CA1 hippocampo-entorhinal projections and nigrostriatal projections.

In the process of thinking about promising mechanisms, we discovered two serendipitous ancillary benefits of copying. The first was that the mechanism of neuronal copying was a neuronal implementation of causal inference [86]. The capacity of STDP to capture temporal relations consistent with causality rather than just correlations has been described by several authors [65], [87], [88], [89]. However, to our knowledge, STDP has until now not been used in an algorithm to explicitly infer whole causal networks. Considerable attention has been paid recently to the capacity of animals such as New Caledonian crows [90], rats [91], non-human apes [91], children [92] and human adults [92] to undertake causal reasoning tasks, i.e. tasks in which good performance cannot be well explained by pair-wise associative learning alone. A Bayesian account can be given of performance in some tasks [93]. Another approach is to give a constraint-based reasoning account that involves graph operations on a Bayes Net and interventions to discover conditional independencies between nodes [42]. Recent work reveals that humans use temporal order, intervention, and co-variation to produce generative models of external events [94]. The STDP-based copying algorithm we describe shares the above features with human causal inference; it infers a dynamical causal model from a set of spike trains that arise from an underlying and invisible causal graph (another neuronal network). If instead this set of spike trains arises from a sensory system, in which the underlying causal graph exists in the outside environment, then the same inference mechanism can be used to produce a neuronal generative model [95] of these external stimuli. Such forward models feature in emulation theories of cognition [96], [97].

Let us critically consider some of our assumptions. We assume that neuron-to-neuron correspondence between layers can be established anatomically perhaps with activity-dependent refinement. However, we do not assume that connectivity information could directly pass between layers. For example (referring to Figure 2), we assume neuron A′ can ‘know’ about A, but we do not assume that the connection between A′ and B′ could ‘know’ about the strong connection between A and B directly by trans-axonal signalling. Such information transmission would obviate the need for a causal inference type algorithm. Although trans-axonal signalling has been identified in the peripheral nervous system [98], no such mechanism in known in the CNS, in which there is reason to believe the task would be much more difficult.

The neuronal implementation of EC1 and EC2 require neurons with the following properties. The neuronal replicator hypothesis predicts their existence. Firstly, they must be capable of computing an XOR function, i.e. 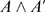 (for EC1) and 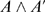 (for EC2) with modifiable temporal windows defined by *T* and *R* respectively. Secondly, they must modulate afferents to A′. EC1 neurons should down-regulate eligibility of synapses to A′ and EC2 neurons should up-regulate eligibility of synapses to A′. This introduces a requirement for non-local plasticity, i.e. spike timing of inputs P, Q at dendrites of neuron X, should be capable of modifying synapses on dendrites of neurons P, Q. Remarkably, Paul Adams had proposed a very similar mechanism of Hebbian *proofreading* that occurs in a canonical neocortical microcircuit [56]. There are Hebbian connections from thalamic neurons to cortical spiny stellate cells. Adams proposes that some deep pyramidal cells (K cells) are coincidence detectors that only reinforce the connection between the thalamic neuron and the stellate cell, if both fire together. Using this, Adams intends to reduce synaptic quantal mutations and prevent an error catastrophe [99]. To modify this mechanism to fit our hypothesis would require the K cells to positively gate all the inputs to the stellate cell when the thalamic input was detected but a stellate cell output was not detected. And vice versa, to negatively gate the high eligibility inputs to the stellate cell when thalamic input was not detected, but stellate output was detected. If this modification to K-cell function is confirmed, our hypothesis predicts that thalamic-stellate cells may represent two layers (L0 and L1), between which copying occurs, i.e. the thalamocortical loop may be a mechanism for running a natural selection algorithm utilizing neuronal copying.

It is an open question what kinds of heritable function neuronal units of selection may possess. At one extreme (the position taken here), function depends on single-neuron connectivity. For example, input I_1_ may pass to A and A′ and output O_1_ may leave from neuron B and B′. The perfect copy with such a rigid input/output reallocation between parent and offspring would need to maintain precise neuron-to-neuron and connection-to-connection identity. However, this is the most difficult case imaginable. At the other extreme, the heritable trait (that co-varies with fitness [100]) is a network property, e.g. the separation property of a neuronal microcircuit [101]. This may mean only the degree distribution of a layer need be copied. The I/O reallocation of inputs and outputs could then be mediated by a single-layer perceptron for example. In general we predict a trade-off in the sophistication of an I/O reallocation device and the demands on fidelity of a copying device.

The functions that natural selection may have in behaviour and cognition are not primary focus of this paper. Candidate functions are category formation [12], global executive control [19]–[21], selective attention using a variant of biased competition theory [102], [103], habit formation [104] and memory reconsolidation [105]. For example Miller and Cohen (2001) write “processing in the brain is competitive: Different pathways, carrying different sources of information, compete for expression in behaviour, and the winners are those with the strongest sources of support” [103]. Desimone & Duncan, (1995) describe how top-down attentional processes bias this competition [102]. Competition is just one aspect of natural selection. We have begun to understand that natural selection undertakes a very powerful kind of heuristic search [29]. Heuristic search may be an algorithm that underlies all the above cognitive functions, in short, it may underlie tasks requiring insight [106].

In which parts of the brain could a natural selection algorithm be implemented? We have relatively little data about the details of cortical circuit topology [45], [107]. Recent developments may allow mapping of microscopic neural circuit topology [108], but in the absence of direct evidence for topology copying we can merely highlight potential candidates. The first possible sites are the loops between medial temporal cortex (including the hippocampus) and the neocortex. . Sirota and Buzsáki (2008) have proposed ‘reciprocal information transfer’ in contrast to the unilateral and passive information–transfer process generally considered [109]. Temporal correlation of the source and recipient neuronal assemblies is a prerequisite for transfer. Topological evolution and copying may occur in these kinds of loops.

Importantly, the loops between the medial temporal cortex (containing the hippocampus) and the neocortex have been implicated in memory consolidation and reconsolidation, processes that involve gradual reorganization of circuits. The “integrative function [of the hippocampus] is taken over by the medial prefrontal cortex” (Fig 1 [110]–[112]) at least for semantic memories. Consolidation has been supported by experimental evidence demonstrating that the anterior cingulated cortex is involved in the remote memory for contextual fear conditioning which is a hippocampus-dependent task [113], [114]. Reconsolidation refers to the process that follows recall. Recall places stable memories in labile and active states. Reconsolidation ensures that the memory is converted back into a stable state and integrated with the recall event [111]. Firstly, topology copying may be involved in this transfer of function from hippocampus to neocortex.

Secondly, we propose that a process of neuronal topology evolution may play a role in the multiple trace theory of consolidation and reconsolidation. Multiple trace theory (MTT) proposed by Nadel and Moscovitch (1997) suggests that complex interactions between the hippocampus and the neocortex including the prefrontal cortex are involved in consolidation and recall of episodic memories [115]. Neuroimaging studies show that when detailed episodic memories are retrieved, the hippocampus is activated [116]–[118]. The MTT proposes that a new hippocampus-dependent memory trace is created whenever an episode is retrieved. The trace may then be strengthened or/and altered and even made more detailed by being linked to additional information from the context. In experiments it has been found that repeated retrieval of the memory caused the memory to be more accessible and more detailed with a concomitant increase in activation within the neocortical regions while activation was maintained within the medial temporal lobe with continued hippocampal activation [119]. Nadel et al (2000) have explicitly proposed that memory traces “decay (i.e. disappear) and can replicate”, in a model that explains some properties of the loss of memory as a function of lesion size [120]. However, there is no description of the internal structure or dynamics of a memory trace.

Some of the features of the hippocampus that may allow memory trace formation include synaptic plasticity, formation of new synapses on dendrites (via stabilization of filopodia in a calcium dependent manner [121]) and unsilencing of silent (AMPA) synapses [122] particularly in conjunction with adult neurogenesis, which is the formation of new (immature) neurons in the dentate gyrus region of the hippocampus [123]. New neurons have been proposed to prevent catastrophic interference of the new memories with existing traces of older memories [124], [125]. The unsilencing of silent synapses, would likewise enable new or modified traces to be formed without interfering with existing traces. The formation of new synapses in a manner based on calcium dependent signalling selecting the survival of a potential synaptic partner formed by a filopodia [121] could be envisaged to allow new contacts to be made between neurons in the same layer.

The second possible site where a natural selection algorithm may be implemented is in the cerebellar cortex. The cerebellum is the site for motor learning. The circuitry of the cerebellum is designed so as to facilitate the learning of a number of contexts by each Purkinje cell. The Purkinje axons are the only output from the cerebellum. Input to the Purkinje neurons are via direct input from the climbing fibres from the Inferior Olive (1∶1 mainly) and via indirect input from the mossy fibres, which originate in the vestibular nuclei and synapse onto the granule cells of the cerebellum. Granule cells make diverging contacts to dendritic arbours of several Purkinje neurons via the parallel fibres. Different views of motor learning have been postulated including the ‘codon representation of an input’ by David Marr in which afferent input events that are communicated by the mossy fibres to the cerebellar cortex are converted into a language of small subsets before being stored [58]. The theory that is most striking is that regarding the possibility of different locations for the memory traces for short- and long-term memories (see [126] for review). Evidence for this theory has recently been found using the horizontal opto-kinetic response (HOKR). The memory for the HOKR has been found to be shunted transynaptically from the cerebellar cortex to the inferior olivary nucleus [127]. A functional memory trace is formed initially within the parallel fibre-Purkinje cell synapses of the cerebellar cortex (flocculus) by an LTD mediated mechanism, and later shunted to the vestibular nuclei (medial vestibular nucleus). There it appears to be consolidated into a long-term memory trace [127]. The mechanism of this shift of the memory from one region of the brain to another is not understood. Because topology copying offers a means of function passing, we propose here that it is a hypothesis worthy of serious consideration.

Neuronal replication, in contrast to DNA replication (Figure 1), is a formidable problem, mainly because the p-bond connectivity in the latter is one-dimensional, in contrast to being two-dimensional in the former. Reproduction of cells is so obvious that the molecular basis of this process has been sought with much energy for a long time. Just by looking at neuronal networks we feel that nothing seems to replicate. No miracle that no efforts have been made so far to identify copying in them. Historically, some people have been fascinated by the algorithm of natural selection producing cumulative adaptations, and postulated that something like that can/must exist in the brain. It has even been said that such a theory can be formulated without a mechanism of neuronal replication, since Darwin did not know the mechanism of inheritance either [32]. Here we aimed at showing how copying in neuronal networks might be feasible. From the proposal it is obvious that evidence for copying would require careful experimentation rather than simple “looking at” networks.

We call attention to the fact that copying of connectivity beyond one dimension has become an issue in replicative nanotechnology as well [128], where the different nodes are identified by unique combinations of linker DNA sequences. Successful replication requires a series of disassembly—copying—reassembly cycles. Neuronal copying is difficult because disassembly is not an option, and template and replica are spatially fixed in position. Yet it now seems that neuronal replication is feasible by sending information through some axons, whereby connectivity implemented by other axons can be copied.
